# Supplementary material for: Influences of dielectric constant and scan rate on hysteresis effect in perovskite solar cell with simulation and experimental analyses
Source: Sci Rep. 2022 May 13;12:7927. doi: 10.1038/s41598-022-11899-x (PMC9106723; doi:10.1038/s41598-022-11899-x)
Supplement: Supplementary file 1 — Supplementary Information 1. [file 41598_2022_11899_MOESM1_ESM.pdf]

Influences of Dielectric Constant and Scan Rate on Hysteresis Effect in Perovskite Solar Cell with Simulation and Experimental Analyses

Jun-Yu Huang,[†] You-Wei Yang,[‡] Wei-Hsuan Hsu,[‡] En-Wen Chang,[†] Mei-Hsin Chen,[‡] and Yuh-Renn Wu[*,†]

†*Graduate Institute of Photonics and Optoelectronics and Department of Electrical Engineering, National Taiwan University, Taipei 10617, Taiwan*

‡*Department of Electro-Optical Engineering, National Taipei University of Technology, Taipei 10608, Taiwan*

E-mail: yrwu@ntu.edu.tw

Section: Details of Experiments

Preparation of Electronic Transport Layer

The SnO_2 colloidal solution was prepared by $SnCl_22H_2O$, the concentration of the precursor solution was adjusted to 0.1 M by adding 25 ml of anhydrous alcohol and 5 ml of deionized water, the SnO_2 colloidal precursor solution suspension was kept stirring for 48 hours.

The TiO_2 precursor solution was prepared by add 3.2 ml of Ethanol into the sample bottle, slowly add 0.8 ml of titanium tetrachloride ($TiCl_4$) to the sample bottle, Rotate at high speed and make sure that the mixed solution is evenly, add a 16 ml Benzyl Alcohol to the hybrid solution of $TiCl_4$ and Ethanol, then placed in the oven heating at 80 °C for overnight. Suck 2 ml TiO_2 precursor solution into a centrifuge tube, add 4 ml ethanol and 2 ml Diethyl Ether, Centrifugation at 5000 rpm for 2 minutes, separate the TiO_2 precipitate from the upper clear liquid, then add 4 ml ethanol and 4 ml Diethyl Ether in the centrifuge tube, this step is repeated twice, and finally, 10 ml of ethyl acetate is added to the centrifuge tube, placed in the ultrasonic cleaning, and shaken evenly to complete the low-temperature TiO_2 solution preparation.

ITO glass substrates were sequentially cleaned with deionized water, acetone, isopropyl alcohol under an ultrasonic cleaner for 15 minutes. After drying the substrate, the substrate is exposed to UV-Ozone for 15 minutes. For the SnO_2 layer, the SnO_2 colloidal solution was spin-coated on ITO substrates at 3000 rpm for 30 seconds, then annealed in air at 180 for 45 minutes; For the TiO_2 layer, the TiO_2 solution was spin-coated on ITO substrates at 2000 rpm for 40 seconds, then annealed in air at 150 for 10 minutes.

Preparation of Perovskite Film

The perovskite precursor solution was prepared in an N_2-filled glovebox by dissolving 578 mg Lead (II) iodide (PbI_2) and 200 mg methylammonium iodide (MAI) in 1 ml dimethylformamide (DMF). The perovskite precursor was spin-coated at 4000 rpm for 20 seconds,

200 μL of chlorobenzene (CB) was dropped on the film at 7 seconds at the beginning of the program, form a uniform thin film. The films were annealed at 100 °C for 10 minutes in an N_2-filled glove box.

Preparation of Hole Transport Layer and Electrode

Precursor solution of the Spiro-OMeTAD was produced by dissolving 80 mg of Spiro-OMeTAD, 28.5 μL of 4-tert-Butylpyridine, 17.5 μL of Li-TFSI solution, in 1 ml chlorobenzene(CB), the Spiro-OMeTAD layer was deposited onto the perovskite film by spin-coating the Spiro-OMeTAD precursor solution at 2000 rpm for 30 seconds in an N_2-filled glove box. Finally, the Au electrodes (thickness 800 nm) were thermally deposited on the Spiro-OMeTAD electron transporting layer, the active perovskite solar cell area of 0.08 cm^2 was defined by the metal mask used during the Au deposition.

Device Characterization

The device performances were measured using a source measurement unit instrument (Model 2420 Source Meter, Keithley Instruments) and a solar simulator (Sun 2000 Class A, ABET technologies) under the standard AM 1.5G (100 mW/cm^2).

Section: FD-TD modeling

The FD-TD algorithm solves Maxwells equation (Eqns. (1)-(2)) in the time and space domain and this method is widely used for optical simulation on nano-structures. The two major Maxwells equations are listed below

$$\nabla \times \vec{E} = -\mu \frac{\partial \vec{H}}{\partial t} \tag{1}$$

$$\nabla \times \vec{H} = \varepsilon \frac{\partial \vec{E}}{\partial t} \tag{2}$$

Here $\vec{E}$ is electric field, $\vec{H}$ is the magnetic field, μ is permeability and ϵ is permittivity. To accurate model wave propagation, it requires a fine mesh, where are large memory is required if we want to model the whole area solar cell. Therefore, to avoid the computation burden, the periodic boundary condition is used. figure S1 is the illustration of optical field modelling domain. First, the AM1.5G solar spectrum is applied. To simplify our modelling, the incident light intensity into the glass substrate was decided by the transmission coefficient at air/glass interface for different wavelength. The reflectance of metal contact at the bottom was also considered. The boundary condition on the top of device is perfectly matched layer (PML) to prevent the problem of repeated reflections from the upper layer. And the period boundary condition (PBC) was utilized on two sides of device (horizon direction). The model had implemented in our previous works.[2,3]

And the simulation result is shown in figure S2. The direction of light incident is from right to left. The calculation domain of Poisson-DD solver is from 0 to 500 (nm), correspond to the Spiro-OMeTAD, $MAPbI_3$ and ETL (SnO_2 or TiO_2). In this FD-TD program, wavelengths of 300 to 800 nm are considered. The result of all spectrum in this work shows in figure S3.

And the all parameters used in FD-TD calculation are shown in Figure. S4.

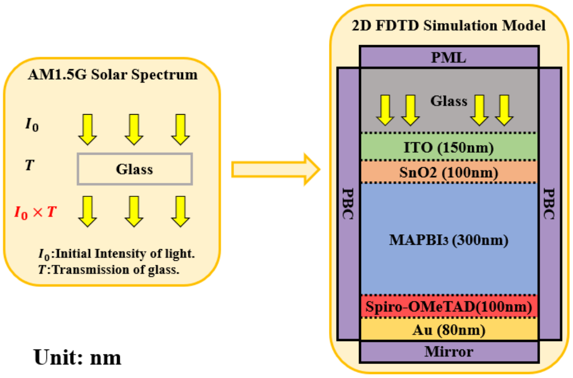

Figure. S1. The illustration of optical field simulation domain, where I_0 is the initial intensity of light and T is transmission of glass that derived from refractive index.

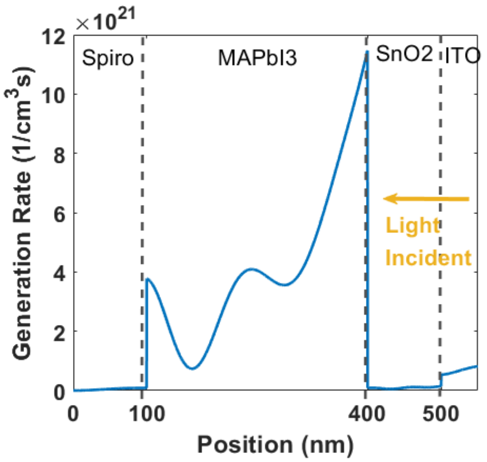

Figure. S2. The generation is calculated by FD-TD method, and it is used in Poisson-DD solver in this work.

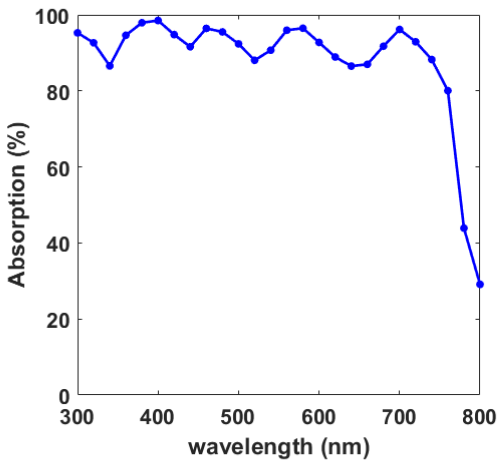

Figure. S3. The absorption from 300 to 800 nm is calculated by FD-TD program.

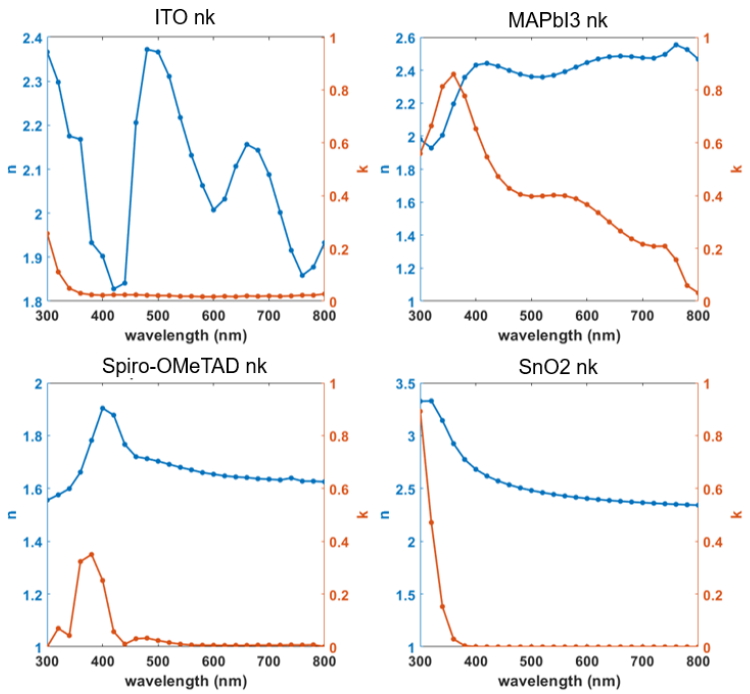

Figure. S4. The refractive index and extinction coefficient for all materials, include ITO, $MAPbI_3$, Spiro-OMeTAD and SnO_2.

Section: Gaussian Density of State

Some characteristics of organic materials, such as the distribution of density of state (DOS) and the field-dependent mobility, differ from those of semiconductors. For the DOS, the distribution of states available for charge hopping can be described by a combination multi-level Gaussian distribution. Therefore, to describe carrier transport in the organic material, the Gaussian distribution function can be introduced as Eqn. (3) to describe the tail states:

$$N_{tail}(E) = N_t \frac{1}{\sigma\sqrt{2\pi}} exp\left[-\frac{(E-E_t)^2}{2\sigma^2}\right], \tag{3}$$

where N_t is the total density of state in Gaussian DOS, σ is the broadening factor of Gaussian shape, the same as standard deviation, E_t is the central position of Gaussian DOS means how deep is this DOS. The schematic figure of Gaussian DOS is shown in figure. S5. In this work, the utilized organic material is Spiro-OMeTAD and the parameters of Gaussian DOS are shown in table. I. And the model had implemented in our previous works.[2,4,5] Also, the

Gaussian DOS is utilized in the perovskite layer to simulate the tail state in perovskite.

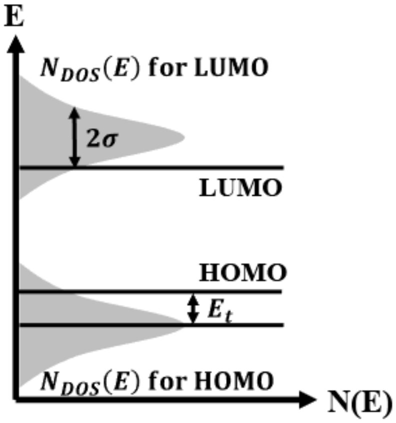

Figure. S5. The schematic of Gaussian density of state in organic materials.

Table 1. Parameters of Gaussian density of states used in this work.

Material	LUMO	HOMO	$N_{t,e}$ (cm^{-3})	$E_{t,e}$ (eV)	$\sigma_{t,e}$ (eV)	$N_{t,h}$ (cm^{-3})	$E_{t,h}$ (eV)	$\sigma_{t,h}$ (eV)
Spiro-OMeTAD	2.10	5.20	1.0×10^{21}	1.95	0.15	1.0×10^{21}	5.3	0.10
$MAPbI_3$	3.82	5.38	1.0×10^{18}	3.82	0.09	1.0×10^{18}	5.38	0.09

Section: Time-dependent Ion Migration Model

To evaluate the effect of the voltage scan rate on hysteresis, the time-dependent ion migration model was coupled with the Poisson-DD solver. The ion migration model considers both the anion (iodide, I^-) and cation (methyl ammonium, MA^+). However, some studies have shown that the mobility of I^- is 10^4 times faster than the mobility of MA^+. Due to the low mobility of MA^+, MA^+ cannot respond to typical scan rates, which range from 10 to 10^4 mV/s. Hence, MA^+ is almost immobile in this model. Therefore, although both types of ions are considered in the model, the net ion density in this work mainly reflects the I^- density, which is shown in figure 1(d) and the net ion density is zero. To evaluate cation and anion migration, the following cation and anion drift-diffusion equations were used:

$$q\frac{dN_{cation}}{dt} = \nabla(q\mu_{cation}N_{cation}E - qD_{cation}\nabla N_{cation}), \tag{4}$$

and

$$q\frac{dN_{anion}}{dt} = \nabla(q\mu_{anion}N_{anion}E + qD_{anion}\nabla N_{anion}), \tag{5}$$

where N is the ion density distribution; μ is the ion mobility; E is the electric field; and D is the diffusion coefficient, which is determined by the Einstein relation $D = \mu k_B T$, where k_B is the Boltzmann constant, and T is room temperature. The subscripts cation and anion indicate MA^+ and I^-, respectively.

The electric profile (electric field, potential and carrier distribution) would be affected by ion migration, then the ion distribution would be affected by electric field at the same time. Hence, this time-dependent ion migration model would couple with Poisson-DD solver until these results are consist in order to model the ion migration effect correctly. And the simulation flow chart is shown in figure S6

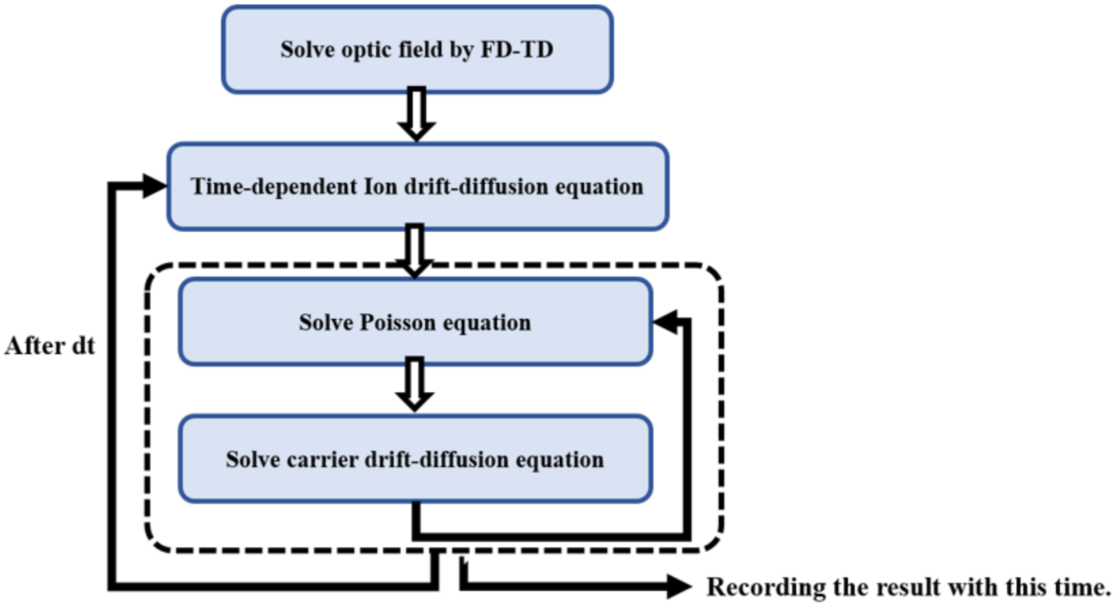

Figure. S6. The refractive index and extinction coefficient for all materials, include FTO, $MAPbI_3$, Spiro-OMeTAD and TiO_2.

Section: Experimental Data - ETL: SnO_2 and TiO_2

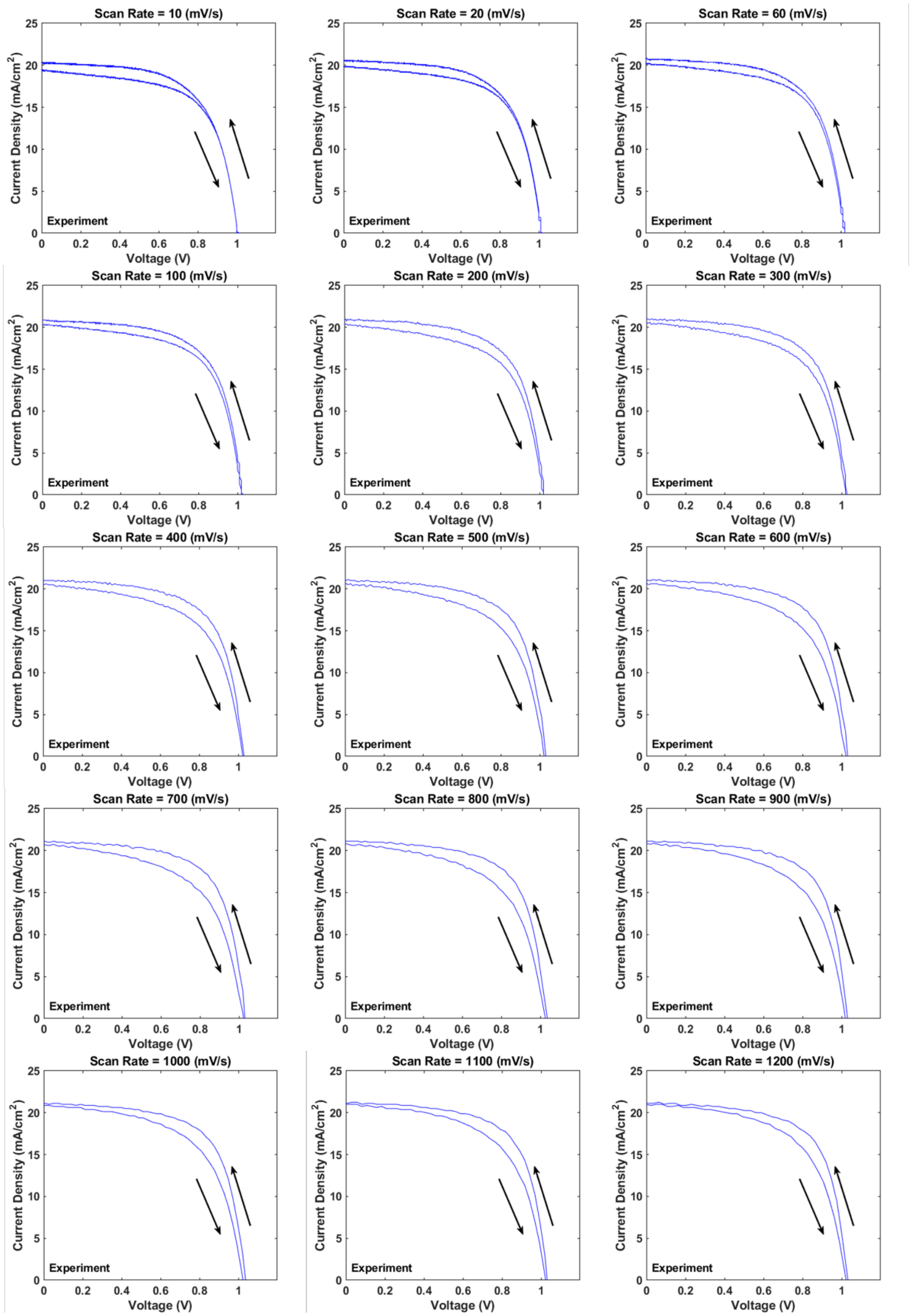

Figure. S7. The experimental J-V curve with scan rate from 10 to 1200 mV/s for case of SnO_2.

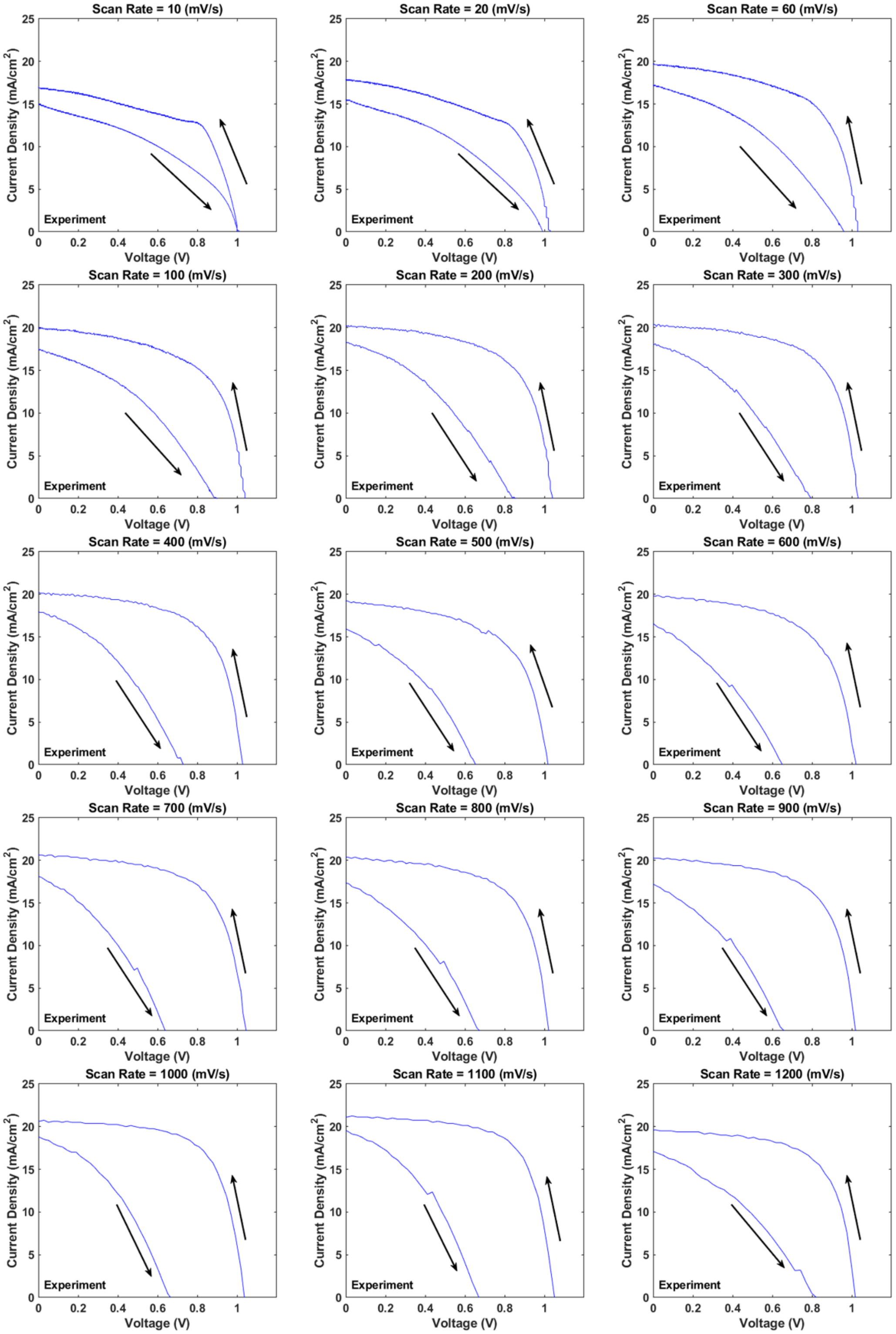

Figure. S8. The experimental J-V curve with scan rate from 10 to 1200 mV/s for case of TiO_2.

Section: The dark Current of Device

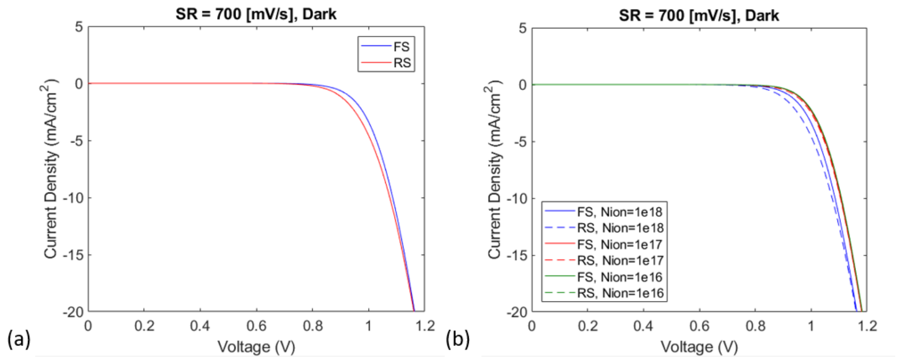

Figure. S9. (a)The J-V curve of case of scan rate being 700 mV/s under dark. (b) The J-V curve with different ion density from 10^{16} to 10^{18}.

References

(1) Lee, J.-W.; Lee, T.-Y.; Yoo, P. J.; Grätzel, M.; Mhaisalkar, S.; Park, N.-G. Rutile TiO 2-based perovskite solar cells. *Journal of Materials Chemistry A* **2014**, *2*, 9251–9259.

(2) Huang, J.-Y.; Chang, E.-W.; Wu, Y.-R. Optimization of MAPbI _3-Based Perovskite Solar Cell With Textured Surface. *IEEE Journal of Photovoltaics* **2019**, *9*, 1686–1692.

(3) Ho, K.-Y.; Li, C.-K.; Syu, H.-J.; Lai, Y.; Lin, C.-F.; Wu, Y.-R. Analysis of the PEDOT: PSS/Si nanowire hybrid solar cell with a tail state model. *Journal of Applied Physics* **2016**, *120*, 215501.

(4) Huang, J.-Y.; Wang, M.-T.; Chen, G.-Y.; Li, J.-Y.; Chen, S.-P.; Lee, J.-H.; Chiu, T.-L.; Wu, Y.-R. Analysis of the triplet exciton transfer mechanism at heterojunction of organic light-emitting diodes. *Journal of Physics D: Applied Physics* **2020**,

(5) Kung, T.-J.; Huang, J.-Y.; Huang, J.-J.; Tseng, S. H.; Leung, M.-K.; Chiu, T.-L.; Lee, J.-H.; Wu, Y.-R. Modeling of carrier transport in organic light emitting diode with random dopant effects by two-dimensional simulation. *Optics Express* **2017**, *25*, 25492–25503.
