# Supplementary figures and images for: Influences of dielectric constant and scan rate on hysteresis effect in perovskite solar cell with simulation and experimental analyses
Source: Sci Rep. 2022 May 13;12:7927. doi: 10.1038/s41598-022-11899-x (PMC9106723; doi:10.1038/s41598-022-11899-x)

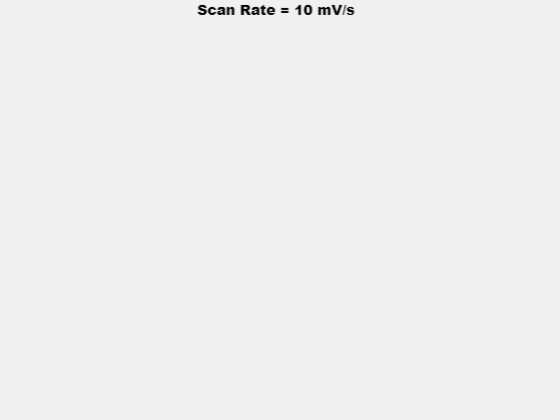

Supplement: Supplementary file 2 — Supplementary Video 1. [file 41598_2022_11899_MOESM2_ESM.gif]

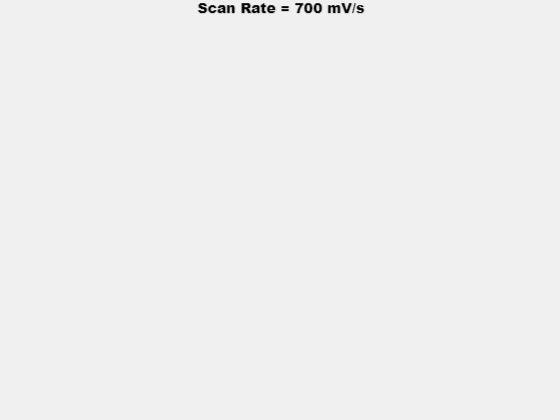

Supplement: Supplementary file 3 — Supplementary Video 2. [file 41598_2022_11899_MOESM3_ESM.gif]

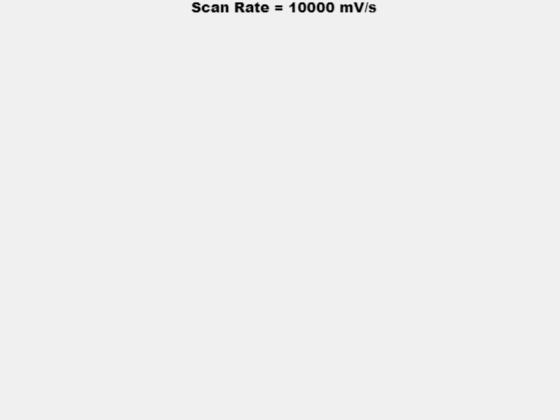

Supplement: Supplementary file 4 — Supplementary Video 3. [file 41598_2022_11899_MOESM4_ESM.gif]
